# Effect of BMI on Mortality in Patients With Tuberculosis and HIV Coinfection in Asia and Africa: Systematic Review and Meta-Analysis

**DOI:** 10.2196/81905

**Published:** 2026-03-03

**Authors:** Mufti As Siddiq M Irzal, Tri Yunis Miko Wahyono, Putri Novia Choiri Insani, Welstin Wemi Loa, Leopardo Alvalius Ngetwa

**Affiliations:** 1 Department of Epidemiology, Faculty of Public Health, Universitas Indonesia, Lingkar Kampus Raya, Jakarta, 16424, Indonesia, 62 895333907275; 2 Department of Nutrition, Faculty of Medicine, Universitas Indonesia, Jakarta, Indonesia; 3 Department of Biostatistics and Epidemiology, School of Public Health, Muhimbili University of Health and Allied Science, Dar es Salaam, United Republic of Tanzania

**Keywords:** Asia, Africa, BMI, HIV, meta-analysis, mortality, tuberculosis

## Abstract

**Background:**

Tuberculosis (TB) continues to pose a major global health threat, particularly in low- and middle-income countries. An estimated 10.8 million people developed TB in 2023, corresponding to 134 cases per 100,000 population. The Southeast Asia region accounted for 45% of global TB incidence, while the African region contributed 24%. Nutritional status, particularly low BMI, is a key modifiable determinant of adverse clinical outcomes. However, its overall impact on mortality among TB-HIV coinfected populations in Asia and Africa remains poorly quantified.

**Objective:**

This study aimed to systematically assess the association between BMI and mortality among patients with TB-HIV coinfection in Asia and Africa.

**Methods:**

A systematic review and meta-analysis were conducted in accordance with the PRISMA (Preferred Reporting Items for Systematic Reviews and Meta-Analyses) 2020 guidelines. Cohort studies published between 2000 and 2024 were identified through PubMed, Scopus, and ProQuest. Data extraction and risk of bias assessment were performed independently by 3 reviewers using the Risk of Bias in Non-Randomized Studies – of Exposure tool. A random effects model with the DerSimonian-Laird method was applied using RevMan 7.2 (Cochrane) to estimate pooled risk ratios with 95% CI. Heterogeneity was quantified using the *I*² statistic, and subgroup analyses were conducted by geographic region.

**Results:**

Seven cohort studies met the inclusion criteria. The pooled estimate indicated that patients with BMI less than 18.5 kg/m² had approximately twofold higher mortality risk compared to those with normal or higher BMI (risk ratio=2.01; 95% CI 1.63‐2.48; *P*<.001). Despite moderate heterogeneity (*I*²=64%), the association remained consistent across subgroups. Although most studies were rated as having high or moderate risk of bias, sensitivity analyses confirmed the robustness of the results.

**Conclusions:**

Low BMI significantly increases the risk of mortality among patients with TB and HIV coinfection in Asia and Africa, underscoring its prognostic and modifiable role in clinical outcomes. Routine nutritional screening, BMI monitoring, and targeted supplementation should be integrated into national TB-HIV management programs, particularly in resource-limited settings. Strengthening TB-HIV nutrition service integration is essential to improve survival and achieve global End TB and United Nations Programme on HIV/AIDS 95-95‐95 targets.

## Introduction

Tuberculosis (TB) and HIV represent 2 converging epidemics that amplify each other’s clinical and epidemiological impact. This syndemic interaction remains one of the most formidable challenges to achieving global health targets. TB and HIV remain major global public health threats, particularly in low- and middle-income countries [1]. The interaction between TB and HIV is synergistically detrimental, as HIV significantly increases the risk of progression from latent TB infection to active disease, while TB accelerates the progression of HIV-related immunosuppression [2,3]. In 2023, an estimated 10.8 million people developed TB globally, an increase from 10.7 million in 2022, reflecting a continued rebound following disruption caused by the COVID-19 pandemic. TB caused an estimated 1.25 million deaths, including 161,000 deaths among people living with HIV. Although mortality has declined by 23% since 2015, this progress remains far below the End TB Strategy milestone of a 75% reduction by 2025 [4]. These figures underscore the urgent need for integrated approaches to meet the World Health Organization End TB Strategy and United Nations Programme on HIV/AIDS 95-95‐95 targets, which emphasize early detection, treatment adherence, and reduction of preventable deaths.

The burden of TB is disproportionately concentrated in low- and middle-income countries, particularly in Asia and Africa, which together account for nearly 87% of global incident cases. A total of 5 countries—India, Indonesia, China, the Philippines, and Pakistan—alone contribute more than half of the global TB burden. The African region continues to experience the highest mortality rate, estimated at 24 deaths per 100,000 population among HIV-negative individuals, compared with only 2.4 in the European region. Coinfection with HIV remains a major driver of mortality, with an estimated 662,000 people living with HIV developing TB in 2023, yet only 58% receiving both TB treatment and antiretroviral therapy (ART) [4].

Several factors exacerbate TB-HIV transmission and mortality. These include socioenvironmental determinants such as poverty, overcrowding, malnutrition, smoking, and exposure to environmental pollutants like biomass fuel and mining dust [5-7]. HIV infection independently contributes to poor nutritional status, which in turn influences TB disease progression and treatment outcomes [8-10]. Compounding this, the COVID-19 pandemic disrupted TB diagnostic and treatment services globally, particularly in Africa and Asia-Pacific, leading to delayed case detection and treatment initiation [11].

Among the modifiable determinants of survival, nutritional status, particularly BMI, plays a crucial role. From a biological standpoint, low BMI reflects impaired nutritional and immunological status, which weakens host defense mechanisms and predisposes patients to severe disease progression and poor treatment outcomes. Undernutrition remains the leading global risk factor for TB incidence, surpassing HIV, diabetes, and alcohol use in its attributable burden. Low BMI (<18.5 kg/m²) has consistently been linked to increased early and all-cause mortality during TB treatment [12]. A prospective cohort study in Ethiopia demonstrated that BMI improvement following initiation of ART was significantly associated with reduced mortality, regardless of initial BMI classification [13]. In contrast, BMI decline within the first month of TB treatment was linked to higher mortality among HIV-positive individuals in Myanmar and Zimbabwe [14]. Additional evidence from Taiwan suggests that underweight male patients have significantly higher TB-specific and non–TB-specific mortality risks during treatment [15].

HIV-associated wasting, often reflected by reduced midupper arm and muscle circumference, further highlights the intersection between nutrition and immune function in TB-HIV management [8,16]. While early ART initiation alongside TB therapy—especially for individuals with cluster of differentiation 4 positive (CD4+) counts less than 50 cells/μl—has been shown to improve survival [17], nutritional assessment and interventions remain underintegrated in TB-HIV care programs, particularly in resource-limited settings.

Despite a growing body of research examining the relationship between BMI and mortality in patients with TB and HIV coinfection, the evidence remains fragmented and inconsistent. Existing studies vary widely in design, sample size, and nutritional cut-off definitions, resulting in heterogeneous findings and limiting the reliability of pooled mortality estimates. Furthermore, no comprehensive systematic review or meta-analysis has been conducted to quantitatively synthesize the magnitude of this association, particularly within high-burden settings such as Asia and Africa, where the dual epidemics of TB and HIV intersect with widespread malnutrition. This lack of consolidated evidence has hindered the development of integrated clinical-nutritional strategies aimed at improving survival among patients with coinfection.

Therefore, this study aims to fill this critical gap by conducting a systematic review and meta-analysis of cohort studies evaluating the effect of BMI on mortality among patients with TB-HIV coinfection in Asia and Africa. This study provides the first quantitative synthesis focusing specifically on Asia and Africa—regions that jointly bear nearly 90% of the global TB burden—offering critical evidence to guide regionally tailored public health interventions. We hypothesize that a lower BMI (<18.5 kg/m²) significantly increases the risk of mortality compared to a normal or higher BMI (≥18.5 kg/m²). The results of this study are expected to provide robust, evidence-based insights to guide targeted clinical interventions and public health strategies for this highly vulnerable population.

## Methods

### Ethical Considerations

This study was conducted using secondary data. This systematic review and meta-analysis is being reported in accordance with the reporting guidance provided in the PRISMA-P (Preferred Reporting Items for Systematic Reviews and Meta-Analyses Protocol) statement [18-20] (Checklist 1).

### Eligibility Criteria

Eligibility criteria for this review included cohort studies (retrospective or prospective) published in the period 2000‐2024 in PubMed, Scopus, and ProQuest databases, with a focus on adult patients (>15 y) with TB and HIV coinfection in Asia and Africa. Only academic journal articles with full-text access were included, while literature reviews, case reports, short reports, proceedings, and studies without mortality data were excluded. The primary outcome evaluated was mortality in populations with TB or HIV coinfection, so studies that did not report mortality rates or examine pediatric populations were excluded from the analysis.

### Data Sources and Search Strategy

A comprehensive literature search was conducted in PubMed, Scopus, and ProQuest to identify relevant studies from 2000‐2024. The search strategy combined Medical Subject Heading (MeSH) terms and free-text keywords related to TB-HIV coinfection, BMI, and mortality. Only studies published in English and involving adult populations (>15 y) were included. Mendeley reference management software (Elsevier) was used to import and maintain the retrieved studies. On October 30‐31, 2024, a search was done to find studies that would be suitable for meta-analysis and systematic review. The full boolean search string used was (“Coinfection”[Mesh] OR “Co-infection” OR “Multiple infection”) AND (“Tuberculosis”[Mesh] OR “TB” OR “Tuberculosis”) AND (“HIV Infections”[Mesh] OR “HIV” OR “Human Immunodeficiency Virus” OR “TB?HIV”) AND (“Adult”[Mesh] OR “Mature” OR “Person over 18”) AND (“Body Mass Index”[Mesh] OR “BMI”) AND (“Mortality”[Mesh] OR “Fatal*” OR “Death” OR “Case fatality rate” OR “Mortality rate”) AND (“Cohort Studies”[Mesh] OR “Cohort study” OR “Cohort”).

### Screening and Selection Process

A total of 3 independent reviewers participated in the methodical and comprehensive screening and research selection procedure. To make it easier to filter titles and abstracts based on preset inclusion and exclusion criteria, all database search results were uploaded to the Rayyan artificial intelligence app (Rayyan Systems, Inc). Before moving on to the full-text screening phase, which was also carried out individually by the 3 reviewers, articles that were found to be duplicates were eliminated. Discussion and agreement were used to settle any disagreements. Data such as author name, year of publication, area, sample size, and number of events in the experimental group (BMI <18.5 kg/m²) and the control group (BMI ≥18.5 kg/m²) were retrieved from publications that satisfied the selection criteria. The data were then recorded in Microsoft Excel spreadsheets to facilitate further management and analysis.

### Risk of Bias Assessment

The risk of bias assessment in this study was performed using the Risk of Bias in Non-Randomized Studies – of Exposure (ROBINS-E) tool, which was specifically developed for evaluating nonrandomized observational studies that investigate the relationship between exposures and outcomes [21]. This tool is particularly appropriate for the present meta-analysis, as all included studies examined the effect of BMI as an exposure on mortality outcomes among patients with TB and HIV coinfection. ROBINS-E assesses 7 domains of potential bias: (D1) bias due to confounding, (D2) bias arising from measurement of the exposure, (D3) bias in selection of participants into the study, (D4) bias due to postexposure interventions, (D5) bias due to missing data, (D6) bias arising from measurement of the outcome, and (D7) bias in selection of the reported result.

Each domain was rated as “low risk,” “some concerns,” or “high risk,” and the overall risk of bias for each study was determined based on the highest level of bias observed across all domains. The use of ROBINS-E was supported by its ability to provide a structured and transparent framework for assessing the internal validity of exposure-outcome relationships in epidemiologic research [22].

### Data Extraction

A total of 3 independent reviewers performed data extraction using a standardized Microsoft Excel spreadsheet developed based on a predefined checklist. Extracted information from each primary study included the author’s name, year of publication, study location, sample size, study design, and the number of deaths and survivors in both groups: the experimental group (BMI <18.5 kg/m²) and the control group (BMI ≥18.5 kg/m²). Any discrepancies between reviewers were resolved through discussion and consensus to ensure data accuracy and consistency prior to quantitative synthesis.

### Outcome Variable and Measures

The primary outcome assessed in this review was mortality among patients with TB and HIV coinfection, measured as the proportion of deaths between the experimental group (BMI <18.5 kg/m²) and the control group (BMI ≥18.5 kg/m²). The measure of association used was the risk ratio (RR) with a 95% CI. For studies that reported odds ratios or hazard ratios, the estimates were converted into RR values using the method proposed by Zhang and Yu (1998) [23], which adjusts for baseline risk in the control group. This standardization ensured that all effect measures were expressed on a comparable scale, facilitating accurate pooling in the meta-analysis.

### Data Synthesis and Analysis

The quantitative synthesis was conducted using Review Manager (RevMan) version 7.2 (Cochrane), applying a random effects model (DerSimonian-Laird method) to account for potential heterogeneity between studies [24]. The pooled effect estimates were expressed as RR with corresponding 95% CI. Heterogeneity was quantified using the *I*² statistic, categorized as low (<25%), moderate (25%‐50%), or high (>50%) [25]. Subgroup analyses were performed based on geographical region (Africa and Asia) to explore potential sources of heterogeneity and assess regional differences in the effect of BMI on mortality. The overall and subgroup pooled estimates were visualized using forest plots.

### Protocol Registration and Deviations

This systematic review and meta-analysis was prospectively registered in the Open Science Framework (OSF.IO/74QWH). Minor deviations occurred between the registered protocol and the final analysis. Specifically, the risk of bias tool was changed from the Hoy tool to the ROBINS-E instrument to better align with cohort study designs. These modifications were made prior to data synthesis and did not affect the overall analytic framework or study objectives.

## Results

### Selection of Studies

**Figure 1.**
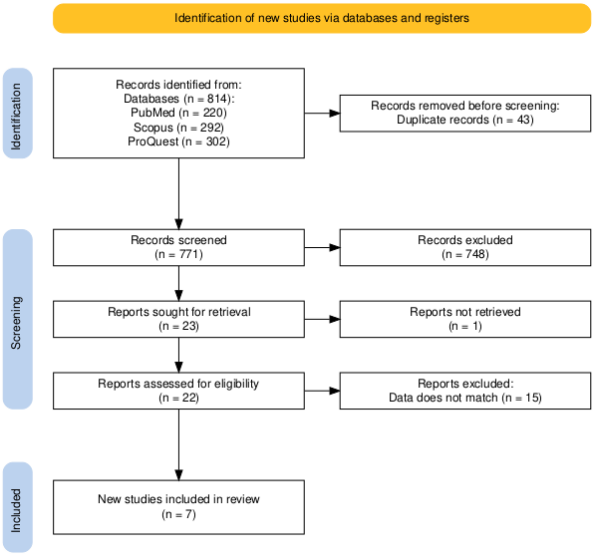
PRISMA (Preferred Reporting Items for Systematic Reviews and Meta-Analyses) flow diagram.

A comprehensive literature search yielded 814 articles from 3 major databases, namely PubMed (n=220), Scopus (n=292), and ProQuest (n=302). After 43 duplicate articles were removed, a total of 771 articles were screened at the title and abstract screening stage, of which 748 were excluded as they did not meet the inclusion criteria. A total of 23 articles were then retrieved for full review, but 1 article could not be retrieved, so 22 articles were assessed for eligibility. At this juncture, 15 studies that failed to meet the requisite data criteria were excluded, resulting in the inclusion of 7 articles in the systematic review and meta-analysis [14,26-32]. The study selection process is detailed in the PRISMA (Preferred Reporting Items for Systematic Reviews and Meta-Analyses) flow diagram (Figure 1).

### Findings

The characteristics of the included studies are summarized by key baseline demographic, clinical, and epidemiological features of TB-HIV coinfected populations across the seven cohort studies. These features include variations in age distribution, sex composition, nutritional status as measured by BMI, HIV clinical stage, CD4 cell count categories, and ART status at baseline. These variables provide essential context for understanding population heterogeneity and for interpreting the pooled mortality estimates derived from the meta-analysis, as detailed in Table 1.

**Table 1.** Data extraction from the included studies.

Author(s) [reference]	Key baseline characteristics^h^	Method	Population/sample	Result
Kegne et al [26]				
	Age distribution: <15 (3.7%), 15-24 (9.5%), 25-34 (36.7%), 35-44 (31.4%), ≥45 (18.7%) Sex: Male (56.6%), Female (43.4%) BMI categories: <18.5 (40.1%), 18.5-24.5 (47.6%), >24.5 (12.2%) WHO^a^ HIV clinical stage: Stage I (6.0%), II (4.7%), III (44.9%), IV (44.4%) CD4^b^ count (cells/µL): <200 (24.2%), ≥200 (75.8%) ART^c^ status/adherence: good (88.3%), fair (7.0%), and poor (4.7%) Variables NR^d^: symptomatic TB status, household TB contact, and viral load	A retrospective cohort study was conducted among patients coinfected with TB^e^-HIV who were treated for TB at public health facilities in Bahir Dar city	The study included 401 patients coinfected with TB-HIV treated between July 2018 and June 2022	Among the 401 patients monitored, 59 (14.7%) passed away during the follow-up period. The research identified several significant predictors of mortality related to BMI. Specifically, patients with a BMI of less than 18.5 kg/m² experienced a mortality risk that was 3 times higher than those with a normal BMI. This finding was substantiated by an aHR^f^ of 3.00, with a 95% CI ranging from 1.44 to 6.28, indicating a statistically significant relationship between lower BMI and increased mortality risk (*P*<.05) [26]
Bayowa et al [27]				
	Age distribution: mean 34.6 (SD 10.5); categories <35 (51.54%), ≥35 (48.46%) ART status at baseline: on ART (95.5%); ART-naïve (4.5%) ART regimen: 1st line (62.1%); 2nd line (4.4%); and 3rd line (33.4%) Viral load categories: detectable (9.7%); undetectable (14.4%); and unknown (75.9%) BMI categories: <18.5 (36.67%); ≥18.5 (63.33%) Variables NR: symptomatic TB status, household TB contact, newly initiated ART, CD4 count, WHO HIV stage	The study used a retrospective cohort design that involved reviewing medical records of patients coinfected with DR-TB^g^ and HIV who were registered at the Mulago Tuberculosis Unit from January 1, 2014, to December 31, 2019	The study included 390 participants who met the eligibility criteria of having confirmed DR-TB and documented HIV positive status, resulting from an initial review of 412 records. Participants were aged from 1 to 80 y, with a mean age of 34.6 y (SD 10.5), and the majority were male participants (53.9%).	The results indicated a mortality rate of 33.2% (95% CI 28.7-38.1) among the study population. Specifically, regarding the relationship between BMI and mortality, a BMI of less than 18.5 kg/m² was associated with an increased likelihood of death. The adjusted incidence rate ratio for those with BMI less than 18.5 kg/m² was 0.91 (95% CI 0.85-0.97), with a *P* value of .007, which suggests a statistically significant protective effect against mortality for individuals with a higher BMI. This highlights the critical importance of maintaining an adequate nutritional status as a determinant of mortality outcomes in this vulnerable population [27]
Gevorgyan et al [28]				
	Age distribution: ≤37 (8.3%); 31-40 (32.8%); 41-50 (32.8%); >50 (26.2%) ART status at baseline: favorable (74.4% on ART); unfavorable (51.1% on ART) CD4 count (cells/mm3): ≤50 (27.6%); 51-200 (18.5%); >201 (17.7%); unknown (55.3%) BMI: underweight (27.4%); normal (45.6%); overweight or obese (8%) Variables NR: Symptomatic TB status, household TB contact, viral load, WHO HIV stage.	The study was conducted in Armenia and used a cohort study design using routine programmatic data of HIV-associated patients with TB receiving treatment from 2015 to 2019.	The population for this research included both pulmonary and extrapulmonary patients with HIV-associated TB, and the study comprised a total of 351 treatment episodes involving 320 individual patients. The authors detailed the sociodemographic and clinical characteristics of these patients through data collected from the National TB database, National HIV database, and patients' medical records.	The results indicated a significant association between BMI and mortality rates among the patients with HIV-associated TB. Specifically, underweight patients had an increased risk of death, reflected in an aHR of 2.5 (95% CI 1.3-4.5), with a *P* value of less than .01. This finding underscores the critical role of nutritional status in influencing health outcomes within this vulnerable population, highlighting the necessity for addressing malnutrition as a part of TB management and care protocols in clinical settings [28]
Kosgei et al [29]				
	Age distribution: 15-24 (11.7%); 25-29 (20.7%); 30-34 (23.8%); 35-39 (20.2%); 40-44 (15%); 45-49 (8.6%). Mean age in years (SD) was 33.3 (7.5) BMI: <15 (12.1%); 15-18.5 (40.8%); 18.5–24.9 (34.1%); >25 (3.1%); missing (9.9%) Time of ART start after TB treatment: <14 days (23.1%); 15-30 days (13.3%); 31–60 days (10.1%); >60 days (7.4%); before TB treatment (29%); not started (17.1%) Variables not reported (NR): Symptomatic TB status, household TB contact, CD4 count, viral load, WHO HIV stage	This study used a retrospective cohort design, analyzing healthcare records from individuals treated for TB between 2012 and 2015. The primary outcome of interest was all-cause mortality during TB treatment.	The study analyzed a total sample of 9026 patients with smear-positive pulmonary TB, aged 15-49 y, who were coinfected with HIV.	The findings revealed that men exhibited a higher mortality rate (11%) compared to women (9%), with a statistically significant difference (*P*=.004). The risk ratio indicated that women had a 17% reduced risk of mortality compared to their male counterparts (aHR 0.83; 95% CI 0.72-0.96; *P*= .013). A higher BMI was associated with a reduced risk of death during treatment; specifically, those with a BMI higher than 18.5 had a lower mortality risk compared to those with a BMI less than 15, underscoring the importance of maintaining a healthy body weight in this population for better treatment outcomes. These results suggest that both gender and BMI significantly influence survival among patients coinfected with pulmonary TB-HIV, highlighting areas for targeted interventions in health care delivery [29]
Naidoo et al [30]				
	Age distribution: median age by BMI groups: 33-36 y (IQR 29-43) ART baseline status: uniform (all initiated ART); detailed categories not reported CD4 count groups: only continuous medians available Viral load categories: only mean viral load reported WHO HIV stage: stage 1-3 and 4 reported by BMI Variables not reported: symptomatic TB status, household TB contact	This study used a retrospective cohort design. Clinical data from HIV-infected patients was evaluated, using statistical methods to assess the correlation between BMI and mortality outcomes. Key analysis tools included multivariate proportional hazards regression to adjust for confounding variables and the log-rank test for comparing survival distributions across BMI categories.	The study comprised a total of 1000 HIV-infected patients. However, due to missing height or weight measurements for 52 patients, the final analysis included 948 patients, split into different BMI categories for assessment. Among these, 389 patients were also coinfected with TB.	The findings indicated that underweight patients (BMI < 18.5 kg/m²) faced a significantly increased risk of mortality compared to those with a normal BMI (18.5-24.9 kg/m²). Specifically, the aHR for mortality in underweight patients was 2.9 (95% CI 1.5-5.7; *P*=.002). This result underscores a notable correlation between low BMI and elevated mortality rates among HIV-infected individuals, highlighting that underweight patients possessed a nearly threefold elevated risk of death relative to their normally weighted counterparts. The study confirms the essential role of baseline BMI as a prognostic indicator, irrespective of TB coinfection status. Notably, mortality rates for those categorized as overweight (BMI 25.0-29.9) and obese (BMI ≥ 30.0) were not significantly different from those with normal BMI, further establishing the critical impact of being underweight on survival outcomes in this population [30]
Wejse et al [31]				
	Age distribution: 15-30 (46%); 30-40 (24%); 40-50 (16%); >50 (14%) All symptomatic; duration reported; asymptomatic NR. HIV status categories: HIV-negative (71.1%); HIV-1 (18.4%); HIV-2 (7.1%); dual (3.4%) CD4 count (cells/mm3): >500 (9.7%); 200-500 (4.9%); <200 (13.3%) Variables NR: Household TB contact, ART baseline status, viral load, WHO HIV stage	The study used a longitudinal, prospective cohort design. Patients were identified through daily visits by field assistants to local health centers and a national referral TB hospital. The diagnosis of TB was made according to WHO criteria, and both smear-positive and smear-negative cases were included based on defined medical and clinical parameters.	The study included a total of 1312 patients with TB who had completed their anti-TB treatment by September 1, 2013. Among these patients, 379 were HIV-infected, comprised of those with HIV-1, HIV-2, and dual infections.	The findings of the study indicated a significant relationship between BMI and mortality among patients with TB. Specifically, the results demonstrated that patients with a BMI less than 17 kg/m² had a crude hazard ratio of 1.42 (95% CI 0.81-2.49), and this association reached statistical significance with a *P* value of .021. This emphasizes that lower BMI is correlated with increased mortality rates in the context of TB management. The study underscores the necessity of monitoring and addressing nutritional status as a critical component of TB treatment protocols [31]
Benova et al [14]				
	Age distribution: median (34-y-old); IQR (29.5-40.2) BMI: <16 (23.6%); 16-18.49 (36.8%); 18.5-24.99 (36.8%); ≥25 (2.8%) ART status at TB treatment start: prior to TB treatment (21.3%); within 2 wk (8.7%); within 2-4 wk (18%); after 4 wk (31.9%); after end of TB treatment (20.1%) CD4 count (cells/mm3): ≤100 (57.8%); 101-200 (21.5%); 201-350 (14.2%); >350 (6.5%) Variables NR: symptomatic TB status, household TB contact, viral load, WHO HIV stage	This retrospective cohort study involved analyzing data collected over 7 y from patients with TB in clinical programs. The study employed Cox proportional hazards regression to evaluate the association between changes in BMI category during the first month of TB treatment and subsequent mortality. Adjustments were made for potential confounders, including sex, age group, project site, and ART status as a time-dependent covariate.	The study included a total of 1090 new adult patients with TB who were HIV-positive, specifically focusing on those diagnosed with smear-negative and extrapulmonary TB. Data were drawn from 3 clinical programs across the mentioned countries.	The analysis revealed a robust association between changes in BMI category during the initial months of TB treatment and mortality outcomes. Patients who remained severely underweight or moved to a lower BMI category had aHR of 4.05 (95% CI 2.77-5.91, *P*<.001) for mortality compared to those who either maintained or moved to a higher BMI category. This indicates a significantly elevated risk of death associated with BMI category deterioration. Similarly, those who remained severely underweight or lost a BMI category showed over twice the rate of unfavorable TB treatment outcomes, with an aHR of 2.53 (95% CI 1.87-3.42). These findings underscore the importance of monitoring BMI changes in identifying individuals at heightened risk of mortality during TB treatment in HIV-positive populations [14]

a WHO: World Health Organization.

b CD4: cluster of differentiation 4.

c ART: antiretroviral therapy.

d NR: not reported.

e TB: tuberculosis.

f aHR: adjusted hazard ratio.

g DR-TB: drug-resistant tuberculosis.

h Note: Absolute frequencies (n) are reported alongside percentages where available. Variations in n values across categories reflect differences in reporting formats and sample denominators in the original studies included in this review.

### Meta-Analysis

In this study, the meta-analysis was conducted using Review Manager (RevMan) version 7.2, using the inverse-variance method under a random effects model to account for possible heterogeneity among the included studies [32]. This analytical approach assumes that the true effect size varies across studies due to differences in study design, populations, and settings, making it suitable for combining data from both Asian and African regions. The DerSimonian-Laird method was used to estimate the between-study variance (*τ*²), providing a more conservative and generalized pooled estimate [24].

**Figure 2.**
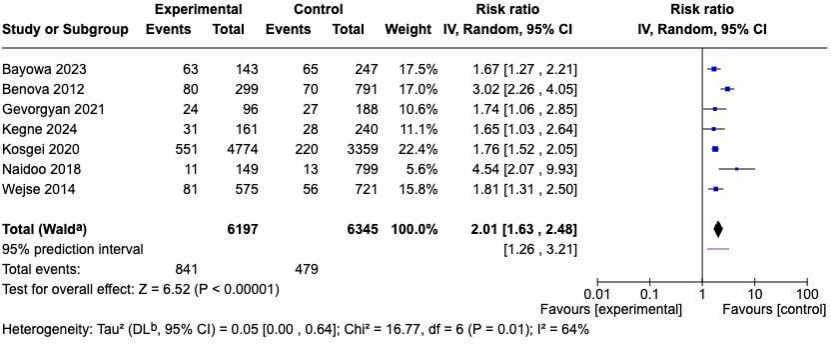
Forest plot of pooled meta-analysis showing the association between low BMI and mortality among patients with tuberculosis and HIV coinfection. The diamond represents the pooled risk ratio estimated using a random-effects model (DerSimonian and Laird method). The 95% CIs were calculated using the Wald-type method [14,26-31].

Effect sizes were expressed as RR with corresponding 95% CI. Statistical heterogeneity was assessed using the *I*² statistic and Cochran *Q* (*χ*^2^) test, which quantify the proportion of total variation across studies that is due to heterogeneity rather than chance [33]. Additionally, a 95% prediction interval was calculated to indicate the expected range of true effects in future studies conducted under similar conditions. The overall results of the meta-analysis are illustrated in the forest plot (Figure 2).

A total of 7 studies involving 12,734 participants (6197 in the low BMI group and 6345 in the normal BMI group) were included in the pooled analysis. The random effects meta-analysis revealed a significant association between low BMI (BMI <18.5 kg/m²) and increased mortality among patients coinfected with TB and HIV. The combined effect estimate indicated that individuals with low BMI had approximately twofold higher mortality risk compared to those with normal or higher BMI (RR=2.01; 95% CI 1.63‐2.48; *P*<.001).

The overall heterogeneity among the included studies was moderate (*I*²=64%; *P*=.01), suggesting that the observed variation in effect sizes was not entirely due to chance. Such heterogeneity was acceptable in meta-analyses of observational studies, particularly given the differences in population characteristics, study design, and regional contexts (Africa and Asia). The estimated between-study variance (τ²=0.05) also indicates that the heterogeneity was not excessive.

**Figure 3.**
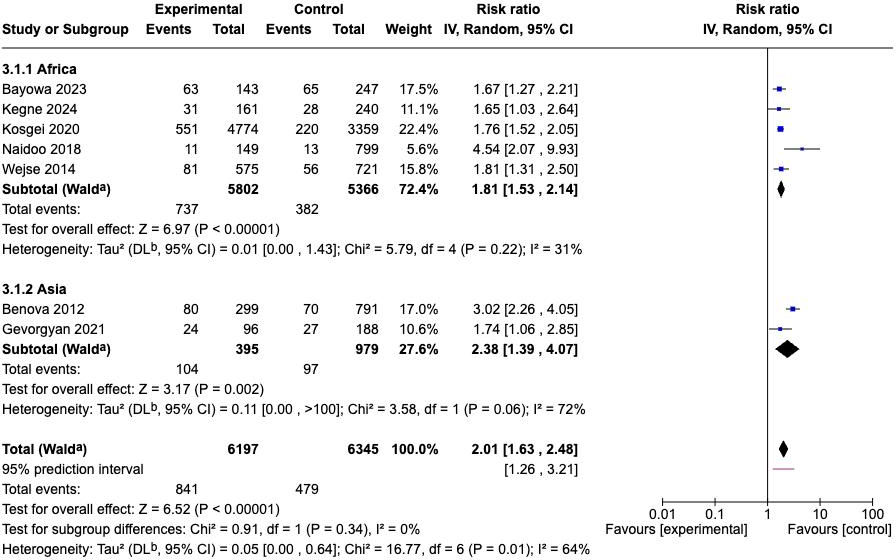
Subgroup analysis of the association between low BMI and mortality by geographic region (Africa and Asia) [14,26-31].

Further, a subgroup analysis by geographic region was conducted to explore potential sources of heterogeneity (Figure 3). The African subgroup yielded a pooled RR of 1.81 (95% CI 1.53‐2.14; *P*<.001; *I*²=31%), indicating a stable and consistent association between low BMI and mortality with low heterogeneity. The Asian subgroup, meanwhile, showed a stronger pooled effect of RR=2.38 (95% CI 1.39‐4.07; *P*=.002; *I*²=72%), reflecting greater variability likely due to smaller sample sizes and study-level differences. However, the test for subgroup differences (*P*=.34) revealed no statistically significant difference between the 2 regional estimates, implying that the detrimental effect of low BMI on survival was consistent across both continents.

These findings provided robust evidence that low BMI was a significant and consistent predictor of mortality among patients with TB and HIV coinfection in both Asia and Africa. The 95% prediction interval (1.26‐3.21) further indicated that future studies conducted under similar conditions were expected to observe a harmful effect of low BMI on survival, even after accounting for between-study variability.

**Figure 4.**
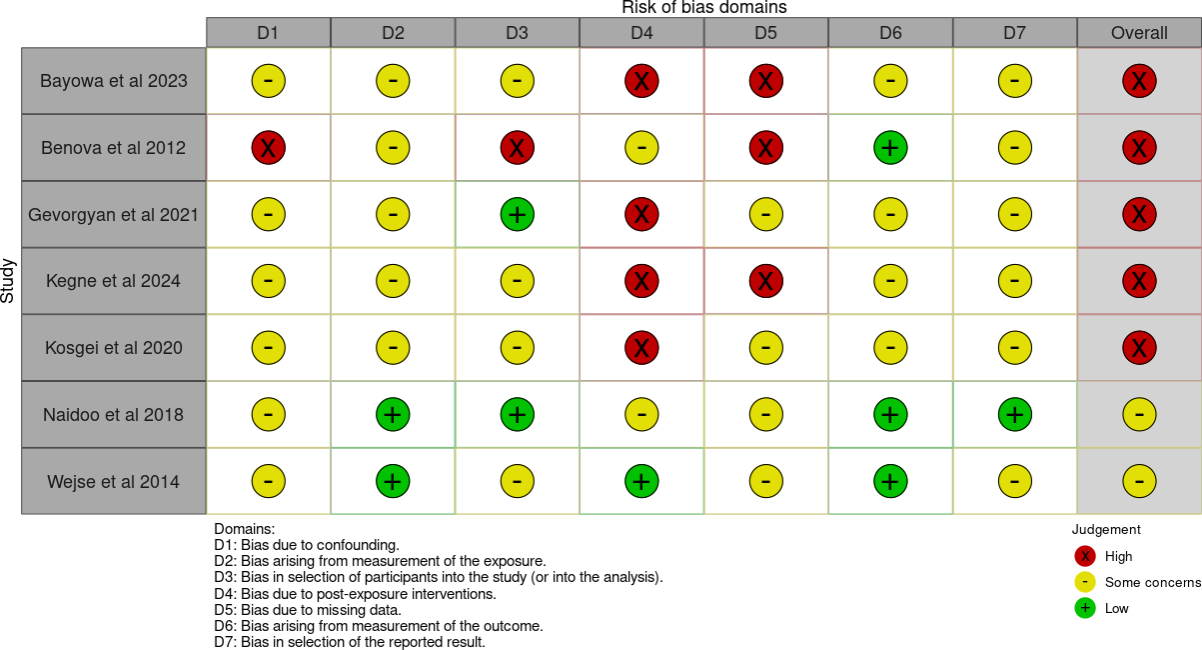
Risk of bias assessment by domain using the risk of bias in nonrandomized studies of exposures tool [14,26-31].

**Figure 5.**
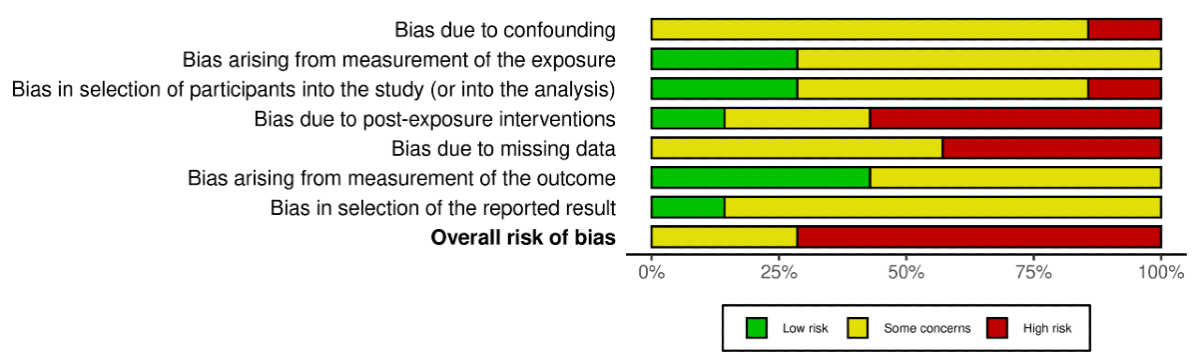
Summary of overall risk of bias distribution across included studies.

To ensure the robustness and internal validity of the pooled estimates presented in both the overall and subgroup analyses (Figure 2), a risk of bias assessment was conducted for all included studies using the ROBINS-E tool. This step was crucial to evaluate potential methodological limitations that might have influenced the observed association between BMI and mortality among patients with TB and HIV coinfection. The detailed assessment across the 7 ROBINS-E domains is illustrated in Figure 4, while the overall summary of bias distribution is presented in Figure 5.

Figure 4 presents the domain-specific assessment of risk of bias for all included studies using the ROBINS-E tool. Most studies showed some concerns in several domains, particularly related to confounding (D1), postexposure interventions (D4), and missing data (D5). A few studies, Benova et al [14] and Kosgei et al [29], demonstrated high risk of bias in multiple domains, mainly due to incomplete adjustment for confounders and lack of clarity regarding data completeness. Conversely, Naidoo et al [30] and Wejse et al [31] exhibited relatively lower risk levels, particularly in domains concerning exposure and outcome measurement. Overall, these findings indicated that while some studies presented notable limitations, the collective evidence base remained methodologically sound for synthesis.

The aggregated distribution of bias levels presented in Figure 5 showed that approximately 60%‐70% of the included studies had some concerns, while a smaller proportion had high risk in at least 1 domain. Importantly, no study was classified as low risk overall, reflecting the inherent methodological limitations typical of observational designs. Despite the predominance of high-risk ratings, all studies were retained for quantitative synthesis because they contributed valuable information and were consistent with the inclusion criteria. Furthermore, sensitivity analysis indicated that exclusion of high-risk studies did not substantially alter the direction of the pooled effect.

In summary, the synthesis of all ROBINS-E domains indicated that the evidence in this review was at moderate to high risk of bias. No studies met the criteria for low risk overall, reflecting the inherent methodological limitations of observational cohort studies in TB-HIV populations, including confounding factors, incomplete baseline information, and heterogeneity in clinical measurements. Nevertheless, the direction and magnitude of the effect estimates remained consistent across studies, and the pooled RR value showed stability in sensitivity analyses. These findings suggested that the association between low BMI and mortality remains strong and credible despite moderate to high risk of bias.

## Discussion

### Principal Findings

The present meta-analysis demonstrated a significant association between BMI and mortality among patients with TB and HIV coinfection in Asia and Africa. The pooled estimate revealed that individuals with BMI less than 18.5 kg/m² had approximately twice the risk of death compared with those with normal BMI. This finding provides strong quantitative evidence that low BMI is an independent predictor of mortality in TB-HIV coinfected populations. The direction and magnitude of this association remained consistent across subgroup analyses by region, further reinforcing the robustness of the effect.

These results corroborate previous observational and cohort studies that have consistently shown that better nutritional status improves survival outcomes in individuals with TB-HIV coinfection. Maintaining or improving BMI during treatment has been associated with faster clinical recovery, reduced incidence of opportunistic infections, and improved immune reconstitution. Conversely, undernutrition compromises immune function, increases susceptibility to severe disease progression, and reduces treatment tolerance, mechanisms that likely explain the higher mortality observed among patients with low BMI.

Subgroup analysis (Figure 3) demonstrated that the association between low BMI and increased mortality remained consistent across both regions, although the magnitude of risk was higher among African cohorts compared to those from Asia. This may reflect regional differences in baseline nutritional status, ART coverage, and health care infrastructure. In Africa, a higher prevalence of advanced immunosuppression at TB or ART initiation and limited access to nutritional support programs likely amplify the impact of malnutrition on mortality. Conversely, in Asian settings, although the overall BMI distribution tends to be higher, undernutrition still poses a significant mortality risk among individuals with HIV-associated wasting and late TB presentation. These findings reinforce the universality of BMI as a prognostic marker, regardless of regional or demographic variation.

For instance, a study in South Africa reported that HIV-infected individuals with a BMI less than or equal to 18.5 experienced significantly higher mortality rates compared to those with a BMI more than 30 kg/m², with corresponding rates of 10.4 and 1.6 per 100 person-years, respectively [34]. Similarly, Yen et al [15] found that patients with TB with a BMI less than 18.5 kg/m² exhibited significantly increased risks of all-cause mortality with adjusted odds ratio (aOR) 1.66, TB-specific mortality (aOR 2.14), and non-TB mortality (aOR 1.58) [15]. Further supporting evidence is provided by studies conducted in Myanmar and Zimbabwe, which demonstrated that remaining severely underweight or experiencing a decline in BMI during the first month of TB treatment significantly increased mortality among HIV-positive patients with smear-negative and extrapulmonary TB [14]. Conversely, in the same South African cohort, overweight and obese HIV-infected individuals had notably lower mortality risks, with adjusted hazard ratios of 0.59 and 0.48, respectively, compared to those with normal BMI [34]. These findings underscore the prognostic value of BMI as an indicator of clinical outcomes in this population.

Malnutrition appears to be a major risk factor contributing to elevated mortality in patients with TB-HIV coinfection. In Taiwan, Lai et al [12] reported that patients with TB with poor nutritional status had a 2.22-fold higher risk of early death within the first 8 weeks of treatment [12]. Likewise, in sub-Saharan Africa, Koethe et al [35] found that low baseline BMI at ART initiation was a robust predictor of early mortality among HIV-infected adults [35].

Several biological and clinical mechanisms may underlie the observed association between BMI and mortality. Low BMI often reflects underlying malnutrition, compromising immune function, including reduced CD4 lymphocyte counts. A CD4 count less than 200 cells/mm³ is widely recognized as a critical threshold of severe immunosuppression, associated with heightened vulnerability to opportunistic infections and increased mortality [36,37]. Additionally, a high HIV viral load (>100,000 copies/mL) reflects impaired immune control and has been linked to increased incidence of TB and mortality risk.

Beyond immunosuppression, malnutrition may exacerbate systemic inflammation. Individuals with low BMI often display a proinflammatory state, characterized by hypercoagulability, endothelial activation, and the development of disseminated intravascular coagulation, a complication associated with poor clinical outcomes [37]. Elevated levels of proinflammatory cytokines and related biomarkers have also been observed in patients with TB and HIV coinfection. They may contribute to tissue damage and multiorgan failure, further elevating the risk of death [38].

In contrast, a higher BMI may provide metabolic and energy reserves that help mitigate the physiological stress of chronic infection. Nutritional status has also been shown to influence treatment efficacy; malnutrition may delay therapeutic response, prolong hospitalization, and worsen overall prognosis [39]. Therefore, comprehensive and individualized nutritional assessments are essential for optimizing treatment outcomes [40]. Furthermore, interactions between food and medications may modulate treatment effectiveness or side effect profiles, reinforcing the bidirectional relationship between nutritional status and treatment success [41].

### Clinical and Public Health Implications

The implications of these findings are substantial for public health strategies. Given the consistent association between low BMI and increased mortality, nutritional interventions should be considered a fundamental component of TB-HIV management protocols. Timely nutritional support—such as the provision of food supplements and access to nutrient-rich diets—alongside early diagnosis may significantly improve clinical outcomes [42,43]. Community-based strategies that incorporate routine nutritional monitoring and adherence support also have great potential to improve survival rates in this population. Evidence shows that food insecurity is a major barrier to adherence in both HIV and TB care, while food baskets and nutritional supplements have been shown to improve treatment adherence and completion [44]. Integrating systematic BMI checks and early assessment of malnutrition risk into routine TB-HIV service delivery will ensure that high-risk patients are identified quickly and that nutritional support is provided as part of standard care, rather than as an additional service.

A coordinated approach linking nutritional rehabilitation with ART initiation and directly observed therapy, short course (DOTS), can further strengthen treatment outcomes. Patients living with TB-HIV who are malnourished often begin therapy with advanced immunosuppression and high vulnerability to early mortality, making nutritional management synchronized with pharmacological therapy crucial. This is particularly important because food insecurity has been shown to worsen ART side effects and reduce adherence when medications are taken without sufficient food [44]. Developing operational guidelines that clearly integrate nutritional support into ART and TB treatment service flows can encourage more consistent and effective implementation across health care facilities.

Finally, at the policy and health system level, integrating structured nutrition services into national TB and HIV guidelines—accompanied by program budget adjustments to maintain food supplementation packages—will be key to long-term sustainability. Strengthening the supply chain for nutrition commodities and including nutrition-related indicators in routine TB-HIV monitoring and evaluation systems can further improve service continuity. These measures offer a viable and sustainable integrated approach to improving TB-HIV coinfection management outcomes in high-burden regions of Asia and Africa, while emphasizing the importance of institutionalizing nutrition-based interventions in the context of resource-constrained health services.

### Conclusion and Recommendations

This systematic review and meta-analysis confirm that low BMI (<18.5 kg/m²) is a strong and independent predictor of mortality among patients coinfected with TB and HIV in Asia and Africa. The pooled estimate (RR=2.01; 95% CI 1.63‐2.48) indicates that individuals with BMI less than 18.5 kg/m² experience approximately twofold higher mortality risk compared to those with normal BMI. This finding reinforces the critical prognostic role of nutritional status in TB-HIV comanagement. Although moderate heterogeneity (*I*²=64%) and variations in study quality were observed, the association between low BMI and increased mortality remained consistent and statistically robust across subgroup analyses.

These findings reaffirm that nutrition forms a cornerstone—not an adjunct—of TB-HIV clinical management. Given the two-fold increase in mortality risk observed among underweight patients, integrating nutritional assessment, intervention, and routine BMI monitoring into TB and HIV programs is essential to improve survival outcomes. National health systems in Asia and Africa should prioritize early nutritional screening and timely supplementation, particularly for underweight patients and those initiating ART. Furthermore, policy frameworks should promote the integration of TB, HIV, and nutrition services through intersectoral collaboration. Future studies and policy evaluations should assess the effectiveness of structured nutrition-ART interventions and explore context-specific BMI thresholds for predicting mortality and enhancing long-term treatment success among TB-HIV coinfected populations.

### Limitations

Several limitations should be considered when interpreting these findings. First, only 7 cohort studies met the inclusion criteria, which may have limited the generalizability of findings to broader TB-HIV populations across diverse clinical settings and precluded stratified analyses (eg, by sex, ART status, or CD4 count). This reflects the scarcity of prospective studies assessing mortality outcomes among TB-HIV coinfected populations in Asia and Africa. Second, substantial heterogeneity (*I*²=64%) was observed, likely arising from differences in study design, clinical settings, ART coverage, and the operational definition of BMI and mortality. Third, variation in BMI categorization and timing of measurement across studies may have introduced misclassification bias. Fourth, although cohort designs support temporal inference, residual confounding remains possible, as critical covariates, such as CD4 cell count, viral load, ART adherence, socioeconomic factors, and comorbidities, were inconsistently reported, potentially leading to residual confounding in the polled estimates. Additionally, none of the included studies reported symptomatic versus asymptomatic TB status, preventing further stratified analyses based on this clinically important variable. Finally, restricting inclusion to English-language publications may have led to language bias and underrepresentation of studies published in local Asian or African journals.

Despite these limitations, this meta-analysis provides robust and consistent evidence that BMI serves as a clinically relevant and modifiable predictor of mortality in patients with TB and HIV coinfection. Strengthening integrated nutritional and clinical care, along with context-specific program implementation, is therefore vital to reduce preventable deaths and to achieve the global End TB and United Nations Programme on HIV/AIDS 95-95‐95 targets.

## Supplementary material

10.2196/81905 Checklist 1 PRISMA (Preferred Reporting Items for Systematic Reviews and Meta-Analyses) checklist.

## Abbreviations

aOR: adjusted odds ratio
ART: antiretroviral therapy
CD4: cluster of differentiation 4
MeSH: Medical Subject Heading
PRISMA: Preferred Reporting Items for Systematic Reviews and Meta-Analyses
PRISMA-P: Preferred Reporting Items for Systematic Reviews and Meta-Analyses Protocol
ROBINS-E: Risk of Bias in Non-Randomized Studies – of Exposure
RR: risk ratio
TB: tuberculosis
WHO: World Health Organization
